# An evaluation of outdoor school environments to promote physical activity in Delhi, India

**DOI:** 10.1186/s12889-016-3987-8

**Published:** 2017-01-05

**Authors:** Samiksha Tarun, Monika Arora, Tina Rawal, Sara E. Benjamin Neelon

**Affiliations:** 1 Saint Louis University School of Medicine, 1402 South Grand Boulevard, St Louis, MO 63104 USA; 2 Health Promotion and Tobacco Control, Public Health Foundation of India, Plot No. 47, Sector-44, Gurgaon, 122002 India; 3 Public Health Foundation of India, Plot No. 47, Sector-44, Gurgaon, 122002 India; 4 Department of Health, Behavior and Society, Johns Hopkins University, 624 N Broadway, Baltimore, MD 21205 USA

**Keywords:** Built environment, India, Physical activity, School

## Abstract

**Background:**

Increasing physical activity in children is an important public health goal in India. Schools may be a target for physical activity promotion, but little is known about outdoor school environments. The purpose of this study was to describe characteristics of the surrounding outdoor school environments that may promote children’s physical activity in Delhi, India.

**Methods:**

For this cross-sectional study, we conducted a structured observation of outdoor school environments in a random sample of 16 private schools in Delhi, India using the Sport, Physical activity and Eating behavior: Environmental Determinants in Young people (SPEEDY) audit tool. The SPEEDY school audit measured six categories, including (1) access to the school; (2) surrounding area; (3) school grounds; (4) aesthetics; (5) usage; and (6) overall environment. Six trained data collectors conducted the audit independently in the summer of 2012 while schools were in session.

**Results:**

Of the 16 schools, one had cycle lanes separated from the road while two schools had cycle lanes on the road. Two schools had pavement on both sides of the road for pedestrians. One school had marked pedestrian crossings. No schools had school warning signs, road safety signs, or route signs for cyclists that would help calm vehicular traffic. Fifteen schools had playground equipment and nine had courts, an assault course (a sequence of equipment designed to be used together), and a quadrangle (an enclosed or semi-enclosed courtyard) for outdoor physical activity. The majority of schools were shielded from the surrounding area by hedges, trees, or fences (*n* = 13) and were well maintained (*n* = 10). One school had evidence of vandalism. Two schools had graffiti, seven had litter, and 15 had murals or art.

**Conclusions:**

The majority of schools did not have infrastructure to support physical activity, such as cycle lanes, marked pedestrian crossings, or traffic calming mechanisms such as school warning signs. However, most had playground equipment, courts, and outdoor play areas. Nearly all were free from vandalism and many had murals or art. These results provide preliminary data for future work examining outdoor school environments, active transport to school, and children’s physical activity in India.

**Electronic supplementary material:**

The online version of this article (doi:10.1186/s12889-016-3987-8) contains supplementary material, which is available to authorized users.

## Background

In India, the growing burden of non-communicable diseases (NCD) is projected to account for nearly 75% of adult deaths by 2030 [1]. Among children in India, the prevalence of overweight and obesity ranges from four to nearly twenty percent [2–6] and is on the rise [7]. Correlates of overweight and obesity among children in India include low levels of physical activity [5, 8, 9], less outdoor physical activity [10–12], lack of athletic ability [13], television and screen time use [5, 11], urban residence [10], and moderate to high family income [4, 5, 13]. Given that insufficient physical activity may contribute to obesity and the prevalence of childhood obesity is increasing in India, intervention efforts are needed to promote physical activity at home, while traveling to school, in school, and in other settings where children spend time [14, 15].

In addition, physical activity in childhood is associated with numerous health benefits [16, 17], as well as continued physical activity into adulthood [18, 19]. Physical activity may also help reduce the incidence of non-communicable diseases in children, which is especially important in low- and middle-income countries because the burden of non-communicable diseases is growing and projected to worsen [1, 20]. Country-level data on physical activity levels of children in India are not available [21], but smaller, regional studies provide some insight. A recent study evaluating physical activity levels in school-age children ages 3–11 years from major cities in India including Bengaluru, Chennai, Hyderabad, Kolkata, Mumbai, New Delhi, and Surat used parent report and child self-report to estimate that 21% of children were inactive, 18% engaged in physical activity at least once per week, 21% engaged in physical activity two to three times per week, and 40% engaged in physical activity more than three times per week [22]. A second study of more than 600 school-age children ages 9–13 years in Mangalore collected self-reported data and found that children living in rural areas spent more time being physically active before school (29 versus 5 min per day) and during school hours (68 versus 27 min per day) compared to their urban counterparts [23]. The studies cited herein indicate room for improvement, and thus highlight the need for additional research to determine effective means to increase children’s physical activity.

Indian guidelines recommend 60 min daily of moderate intensity physical activity for school-age children [24]. These guidelines also encourage physical activity while children are in school, especially because opportunities at home may be lacking. Children often spend over six hours per day at school, and time at home is typically spent doing homework, watching television, or using electronics [25, 26]. Thus, school may be the primary setting for children’s physical activity. It is important to assess the school environment and evaluate opportunities to promote physical activity [27–29].

Interventions promoting physical activity in schools could target outdoor school grounds. Previous studies have included outdoor environments in successful school-based interventions in other countries [30–32]. Little is known, however, about school environments in India and results from these studies may not be culturally relevant to low-income countries such as India. Despite the lack of knowledge, we believe it is a logical extension to build on this previous work and determine if there is any feasibility of these concepts in the Indian context. To our knowledge, only two previous interventions targeted school-age children in India. Saraf et al. [33] evaluated a multi-component intervention, which consisted of giving study participants information about school policies, physical activities and more to prevent non-communicable diseases in 40 middle schools in rural Ballabgarh, North India. They found improvements related to physical activity—more intervention schools adopted physical activity policies and student participation in physical training classes increased significantly in intervention compared to control schools [33]. Next, two schools in Pune and Nasik implemented a five-year school-based intervention that consisted of daily yoga, breathing exercises, physical activity lessons taught in school, healthier school meals, and health and nutrition education for teachers, students, and their families [34]. Results from the study showed that children in the intervention group were more fit than controls in running, long jump, sit-up, and push-up tests. Other benefits in the intervention group included less time in sedentary activities and more time in active play and healthier eating of fruits. However, there was no difference in body mass index or prevalence of overweight or obesity levels between intervention and control groups. The benefits indicate that school based interventions have a potential for increasing physical activity, and it would be interesting to see this applied to school grounds. However, with only a few studies to provide insight, additional research is needed to assess the suitability for physical activity interventions targeting school-age children in India. In this study, we used the SPEEDY school audit to measure six different categories to capture aspects of the school grounds, including (1) access to the school; (2) surrounding area; (3) school grounds; (4) aesthetics; (5) usage; and (6) overall environment. The purpose of this study was to describe and analyze characteristics of outdoor school environments that may be future intervention targets to help promote children’s physical activity in Delhi, India.

## Methods

### Study design and sample

For this cross-sectional study, we conducted a structured observation of outdoor school environments in a randomly selected sample of schools in Delhi, India. To select our sample of schools, we first obtained a list of all schools recognized by the Directorate of Education (DOE), Government of National Capital Territories of Delhi [35] in the twelve districts of Delhi, including primary, middle, secondary, and senior secondary schools. We excluded schools that were publically (government) funded, as we could not obtain permission from the DOE to conduct our assessments. Instead, we focused on the 1297 private schools, as these schools received little to no financial assistance from the government. We assigned each qualifying school a number, and then randomized that list. From that randomized list of 1297 schools, we selected the first 50 schools to contact as a convenience sample. We excluded two schools that did not have outdoor school grounds. Of the remaining 48 schools, 16 school administrators agreed to participate in the study. Private schools in India do not publically report the mean age or number of children in attendance. The only information publically available or shared with the research team was that children enrolled in the schools ranged in age from 5 to 17 years. Because we did not include data on children, the Institutional Review Boards of Duke University and the Public Health Foundation of India granted a waiver for ethical approval for this study.

### Measures

The Sport, Physical activity and Eating behavior: Environmental Determinants in Young people (SPEEDY) school grounds audit tool was developed by researchers at the University of Cambridge as part of a large longitudinal study examining physical activity in 9- to 10-year-old school children [36, 37]. The SPEEDY school audit measures outdoor school grounds and can be scored to quantify environmental support for physical activity. The audit was developed through modification of existing tools designed to assess the quality of urban green spaces, as well an existing audit tool of preschool playgrounds [36, 38, 39]. The developers evaluated the reliability and validity of the audit tool in 92 primary schools in England [36]. They found reliability and validity to range from acceptable to good, and higher scores on the audit tool scores correlated with higher levels of children’s physical activity within schools.

We adapted the school audit tool for use in India. A panel of nine school-based experts and Delhi residents provided input on the relevance of each item on the tool. We did not add or remove any items from the tool. We did insert an “additional comments” item in the user manual that included space for qualitative comments from data collectors in case issues arose in the field that required later discussion among the research team.

The SPEEDY audit tool consists of 39 items, and includes six categories: (1) access to the school; (2) surrounding area; (3) school grounds; (4) aesthetics; (5) usage; and (6) overall environment. The category ‘access to the school’ consists of two items that record the entrances for cars, pedestrians, and cycles; it also determines whether there is a speed limit and roadside parking available for each entrance. The ‘surrounding area’ is defined as the area visible from any of the entrances of the school. This category consists of 12 items and focuses on the presence or absence of various factors such as cycle lanes, areas where parents can drop off children, a paved bus stop, marked pedestrian crossings, traffic calming, and various road signage visible from any of the school entrances. The ‘school grounds’ category consists of a total of 13 items, including playground equipment, assault courses (i.e., sequence of pieces of equipment designed to be used together), quadrangles (i.e., enclosed or semi-enclosed courtyards), pitches (i.e., marked areas typically for sport with an incline and associated goal or flag), athletic tracks, courts, benches, picnic tables, wildlife gardens, uncovered and covered cycle parking, and water coolers. The ‘aesthetics’ category consists of six items evaluating as trees, bed, and other vegetation on school grounds, ambient noise, litter, and murals. The ‘usage’ category assesses, in three questions, whether the school grounds are suitable for sport, games, and general play. The ‘overall environment’ includes three items to determine whether the school grounds are shielded from the surrounding area by protective borders such as trees, fences, and hedges, and if the grounds are well maintained and generally free of vandalism. Additional information about the SPEEDY school audit is available in Additional file 1 and elsewhere [36].

We coded the school audit consistent with the approach used by its developers [36]. For the twelve dichotomous items in the ‘surrounding area’ category, we used a binary code of ‘0’ (item not present) and ‘1’ (item present). We assessed potential qualities of ‘good’, ‘average’, or ‘poor’ for each item in the category of ‘school grounds’. For the quality, we reported the median value. Because there was an even number of schools, the median may be represented with two quality descriptors (e.g., good/adequate). We coded items in ‘aesthetics’ and ‘usage’ with three response options (none, some, or a lot; not at all, somewhat, or very; poor, adequate, or good) as ‘0’, ‘1’, and ‘2’, respectively. Three questions in the ‘overall environment’ category included a five-point likert scale response option, ranging from ‘-2’ to ‘2’. Thus, positive values were considered more beneficial. We applied reverse coding to three items: noise, litter, and graffiti. We analyzed audit data using STATA 12.0 (Stata Corp, College Station, Texas, USA, 2012).

### Data collection

Six trained data collectors conducted school audits. Each data collector participated in a 5-h training session prior to conducting the audits. To assess inter-rater reliability, data collectors completed the audit tool independently at the same school; we assessed agreement for each item on the school audit tool. We calculated kappa test statistics and found moderate to high agreement among data collectors for each category (range: 0.4-1.0) on the school audit, with an overall agreement among raters of 76.6%. The lowest agreement was for ‘school grounds – quality’ (61.9% agreement) and ‘aesthetics’ (61.9% agreement), while the highest was for ‘surrounding area’ (100.0% agreement). All audits were completed during school hours while school was in session Monday through Saturday 8:00 am–3:30 pm. We collected data during the summer of 2012 (June–August), while school was in session in Delhi. Data collectors completed the audits independently, but were sometimes accompanied by a school official.

## Results

The 16 schools that participated in the study were located in nine of the 12 districts in Delhi. Of the 16 participating schools, 37.5% (*n* = 6) were located in a residential area, 18.8% (*n* = 3) were in a business or retail building area, 18.8% (*n* = 3) were in a mixture of business and residential areas, 12.5% (*n* = 2) were in open fields or parks, one school was surrounded by a hospital, and one was surrounded by a combination of these settings. The ‘access to school’ category was used for data collectors to orient to the school grounds and determine the various components of the school grounds that would be further evaluated with the tool.

### Surrounding area

Fourteen of the 16 schools (87.5%) had space for parents to drop off or pick up children and a place for parents to park their cars (Table 1). Ten schools (62.5%) had a school bus stop. Two (12.5%) had cycle lanes on the road, pavement on both sides of the road, and a marked pedestrian crossing. One school (6.3%) had pavement on one side of the road only and one had cycle lanes separated from the road and marked pedestrian crossing. No schools had school warning signs, road safety signs, or route signs for cyclists.

**Table 1:** Percent of schools with ‘surrounding area’ items in Delhi, India

‘Surrounding area’ items	Percent (number) of schools
Space for parents to stop and drop off or pick up children	87.5 (14)
Somewhere where parents can park their cars	87.5 (14)
School bus stop	62.5 (10)
Cycle lanes separated from road	6.3 (1)
Cycle lane on the road	12.5 (2)
Pavement on both sides of the road	12.5 (2)
Pavement on one side of the road only	6.3 (1)
Marked pedestrian crossing	6.3 (1)
Traffic calming	25.0 (4)
Signage: school warning signs for road users	0.0 (0)
Signage: road safety signs	0.0 (0)
Signage: route signs for cyclists	0.0 (0)

### School grounds

Fifteen schools (93.8%) had playground equipment and ten (62.5%) had water coolers (Table 2). Nine schools (56.3%) had uncovered cycle parking, assault courses, and quadrangles. Seven schools (43.8%) had pitches, six (37.5%) had athletic tracks, five (31.3%) had benches, four (25.0%) had chalk markings on play surfaces, two (12.5%) had wildlife gardens, one (6.3%) had covered cycle parking, and one (6.3%) had a picnic table. We scored the item examining the presence of courts with a median quality of ‘good’, while all other items were ‘adequate’. We scored the question assessing benches with a median quality of ‘good/adequate’ (since the median value incorporated the two), while wildlife gardens received a median quality of ‘adequate/poor’. One school had all 13 of the ‘school grounds’ items, one school had ten items, and the lowest school had only one item.

**Table 2:** Mean number of items per school and quality of ‘school grounds’ items in Delhi, India

‘School grounds’ items	Mean number (standard deviation) of items per school	Percent (number) of schools with item present	Median quality
Chalk powder markings on play surfaces	0.8 (1.9)	25.0 (4)	Adequate
Playground equipment	5.1 (4.3)	93.8 (15)	Adequate
Pitches (marked area with an incline with an associated goal or flag)	0.8 (1.2)	43.8 (7)	Adequate
Athletic tracks	0.6 (1.0)	37.5 (6)	Adequate
Courts	1.1 (1.3)	56.3 (9)	Good
Benches	1.6 (2.9)	31.3 (5)	Good/Adequate
Picnic tables	0.6 (2.3)	6.3 (1)	Adequate
Water coolers	5.0 (6.2)	62.5 (10)	Adequate
Wildlife gardens	0.1 (0.3)	12.5 (2)	Adequate/Poor
Uncovered cycle parking	6.2 (8.9)	56.3 (9)	Adequate
Covered cycle parking	7.6 (30.3)	6.3 (1)	Adequate
Assault course (sequence of pieces of equipment designed to be used together)	0.7 (0.8)	56.3 (9)	Adequate
Quadrangle (enclosed or semi-enclosed courtyard)	0.8 (0.7)	56.3 (9)	Adequate

### Aesthetics

Fifteen of the 16 schools (93.8%) had ‘a lot’ or ‘some’ murals or outdoor art present (Table 3). Thirteen schools (81.3%) had planted beds and eight (50.0%) had trees (either ‘a lot’ or ‘some’ for both items). Two of the 16 schools (12.5%) had graffiti (‘a lot’ or ‘some’) and seven (43.8%) had ‘some’ litter. Five schools (31.3%) had ‘a lot’ of ambient noise. Three schools had murals, trees, and planted beds (excluding the “negative” items of ambient noise, litter, and graffiti), followed by four schools with two of the items and four schools with one.

**Table 3:** Percent of schools with items in ‘aesthetics’, ‘usage’, and ‘overall environment’ categories in Delhi, India

	Percent (number) of schools with item present		
‘Aesthetics’ items	A lot	Some	None
Planted beds	6.3 (1)	75.0 (12)	18.8 (3)
Trees to sit under	6.3 (1)	43.8 (7)	50.0 (8)
Ambient noise	31.3 (5)	43.8 (7)	25.0 (4)
Litter	0.0 (0)	43.8 (7)	56.3 (9)
Murals or outdoor art	68.8 (11)	25.0 (4)	6.3 (1)
Graffiti	6.3 (1)	6.3 (1)	87.5 (14)
‘Usage’ items	Very suited	Somewhat suited	Not at all suited
Sport	25.0 (4)	50.0 (8)	25.0 (4)
Informal games	31.3 (5)	56.3 (9)	12.5 (2)
General play	37.5 (6)	50.0 (8)	12.5 (2)
‘Overall environment’ items	Agree or strongly agree	Neutral	Disagree or strongly disagree
Grounds shielded from surrounding area	81.3 (13)	0.0 (0)	18.8 (3)
Grounds generally well maintained	62.5 (10)	18.8 (3)	18.8 (3)
Grounds generally free of vandalism	93.8 (15)	0.0 (0)	6.3 (1)

### Usage

Four of the schools (25%) were ‘very suited’ and eight were ‘somewhat suited’ (50%) for sport (Table 3). Five (31.3%) were ‘very suited’ and nine (56.3%) were ‘somewhat suited’ for informal games. Fourteen schools (87.5%) were either ‘very suited’ (*n* = 6) or ‘somewhat suited’ (*n* = 8) for general play.

### Overall environment

The majority of schools were shielded from the surrounding area by hedges, trees, or fences (81.3%, *n* = 13), well maintained (62.5%, *n* = 10), and generally free of evidence of vandalism (93.8%, *n* = 15) (Table 3).

## Discussion

In this study evaluating outdoor school grounds in Delhi, India, we found that the majority of schools were lacking in important surrounding area infrastructure that might encourage physical activity, such as cycle lanes, marked pedestrian crossings, and traffic calming measures. Nearly all schools in our study had an area for parents to drop children off or park their cars to bring children to school. However, few had traffic calming measures or marked pedestrian crossings. Based on studies in high-income countries, walking and cycling to school may increase physical activity levels of children [32, 40–42]. One study in the United Kingdom showed that providing road safety features, such as cycling infrastructure or a crossing guard to allow for safe crossing of the street, helped to support maintenance of moderate to vigorous physical activity levels in school-age children and prevent sedentary behavior [43]. Despite there being road safety guidelines to be followed around schools in Delhi, the lack of surrounding area infrastructure found in our study indicates a need for increased safety measures for walking and biking within the outdoor school environment to encourage physical activity in and around school. Studies have shown that creating a more walkable environment may yield higher levels of physical activity and less automobile driving, which can impact rates of obesity [27, 42]. Creating a more walkable environment around schools by increasing road safety features, like safe crossing of streets with crossing guards, is an important policy implication of this research.

Additionally, in this study we found that the built environment surrounding schools within an urban city can consist of businesses and retail, residential living, open fields or a combination. The surrounding built environment likely affects the walkability of the neighborhood around the school [44, 45]. An environment that is heavily filled with businesses and retail is likely to be less walkable and have more vehicular traffic, while an environment that has open fields or residential areas will likely be more walkable and have less traffic. These variables could affect active travel to and from school among children [46].

The majority of schools had playground equipment, courts, quadrangles, and playground equipment designed to promote physical activity. Results from the school grounds assessment in our study were somewhat comparable to the United Kingdom study where the SPEEDY school audit was developed [36], despite the distinct settings. Compared to the United Kingdom study where 95.7% of schools had courts (versus 56.3% in our study), 75% had pitches (versus 37.5%), and 73.9% had athletic tracks (versus 43.8%) [35]. The mean values for chalk powder markings, athletic tracks, and assault course, however, were very similar between the two studies [36]. Additionally, many of the schools in our sample in India had no items except playground equipment. Brightly colored playground equipment could be a way to promote physical activity among school children [47], but additional studies are needed to assess their impact in schools in India. Overall, schools in our study in India had substantial room for improvement compared to schools in the United Kingdom.

Outdoor play equipment may be especially important in promoting school-age children’s physical activity. One study conducted in the United States found that when children were given free choice within an outdoor play area, they were more likely to spend time in areas with playground equipment and less time in open fields [48]. Another study of schools in New Zealand found an association between a greater number of fixed play equipment on school grounds and higher levels of physical activity in children [49]. Thus, outdoor play equipment may increase children’s physical activity at school [50], although there is some evidence that girls are more likely than boys to utilize playground equipment [51]. However, given that these previous studies were conducted in high-income countries, additional information is needed to evaluate outdoor play equipment and children’s physical activity within a low-middle income country like India.

We also found that nearly three quarters of schools in our sample had outdoor grounds that were ‘somewhat suited’ for sport, informal games, and general play. Additionally, more than half had planted beds and murals or outdoor art. Half of the schools also had trees that students could sit under. The overall environment of the school grounds was strongly positive because most were shielded from the surrounding area, generally well maintained, and mostly free of vandalism. Few schools had graffiti, although a large number had some litter present. Prior studies have shown that aesthetics within the environment such as signs, artwork, and music can promote physical activity—especially on outdoor playgrounds [31, 43, 49–51]. A previous intervention study in the United Kingdom introduced colorful playground equipment and found increased physical activity levels of children in schools [43]. In a similar study in the United Kingdom, multicolor playground markings increased activity levels of school-age children [51]. However, it is not clear if these results would be similar in a low-middle income country like India. Future studies should assess the extent to which outdoor aesthetics in schools in India are associated with physical activity in children.

Physical activity and built environment research are relatively new domains of research in India and in other low- and middle-income countries. To our knowledge, no previous study thus far has evaluated the school built environment as a potential target to promote physical activity in children in resource-limited settings. However, a recent review of 19 interventions to prevent obesity in low-income countries, including some that aimed to increase physical activity, found that the majority were effective in reducing body mass index, decreasing sedentary behaviors, and increasing physical activity [52]. Another study compared physical activity and sedentary behavior among school children from 34 countries and found that few engaged in sufficient physical activity according to country standards and recommendations. Indian children, while still relatively low, had the highest prevalence with just over one third of children meeting national physical activity recommendations [53]. In a qualitative study of 4^th^ and 5^th^ grade students in Indian schools, children reported moderate awareness of the health benefits of physical activity [54]. Thus, children in India may already have some understanding of the importance of physical activity but may require interventions to create healthier environments to support this behavior. India has recently adopted a multi-faceted action plan for NCD prevention and control. This research and suggested future studies can guide an ideal school environment to promote physical activity among students. In consultation with Department of Education, the Ministry of Health and Family Welfare can use this evidence to develop guidelines to promote physical activity through schools.

Based on this study, we recommend a multi-pronged policy approach to help promote physical activity among school children in urban centers in Delhi, India. Many stakeholders such as government officials, law enforcement, teachers, school administrators, and parents need to collaborate to achieve this policy goal. Government officials need to pass policy that reflects an understanding and ensures that areas surrounding schools have safe infrastructure for students to walk and cycle. Law enforcement officers can help ensure that vehicles follow speed limits and honor stop lights and stop signs, which will help ensure children’s safety when walking and cycling on the surrounding streets. Teachers and school administrators can support curricula that include time for physical activity. There also needs to be some monetary investment in better school grounds equipment, maintenance of school grounds, and continued space for physical activity. Finally, parents need support and encouragement to serve as advocates of physical activity on behalf of their children and role model physical activity behaviors, especially since parental weight status is linked to that of their children [55, 56].

There are a number of obstacles to promoting school-based active travel in low-and-middle income countries. One potential hurdle that needs to be addressed is that there are many stakeholders involved but no one impetus that will drive change. Thus, there are many observations made but not necessarily any concrete steps taken for change. Despite listing this multi-pronged policy recommendation, all policy implications need to be evidence-based and context-specific. Thus, we advocate for more robust research in this field to better understand and evaluate appropriate policy changes to improve school grounds and the surrounding built environment to improve physical activity amongst school-aged children. We also think it may be worthwhile to conduct research in developing local and national programs that promote physical activity and active transport to schools such as “Safe Routes to School” as seen in high-income countries [57].

Our study has a number of limitations. First, we conducted the study in a small sample of private schools located in Delhi. Private schools may be different from public schools in Delhi, and additional information is needed to assess and compare their outdoor school environments. We also did not have information on the number of children enrolled in each school or any additional demographic information about the children (e.g., socioeconomic status) beyond the age range of 5 to 17 years. Thus, our results are potentially limited in their generalizability to other private schools in Delhi. We also focused our assessments on outdoor school environments, and did not evaluate the indoor environments of schools. It is possible that schools in Delhi may have areas within schools for children to be physically active, and these spaces could vary considerably by school and also between public and private schools. Additionally, while the developers evaluated the SPEEDY school audit previously, we did not conduct additional reliability or validity assessments in our sample of schools in India. A previous study modified a neighborhood physical activity assessment developed for use in the United States; the researchers modified the tool for use in India and a few countries in Africa and conducted extensive reliability testing to ensure suitability [45]. As we did not conduct any reliability testing prior to implementation of the SPEEDY school audit, we are not able to fully evaluate the robustness of the data collected using the tool. Finally, we did not conduct any child-level assessments, such as measuring children’s levels of physical activity using accelerometers and the location of that activity using portable global positioning system (GPS) devices, as is the gold standard. Future studies could explore the extent to which supportive school environments encourage physical activity in children, examining both the outdoor and indoor school environment and the corresponding physical activity levels of children. Thus, results from our study can be considered exploratory, and findings may be used to generate hypotheses and stimulate future research in this area.

## Conclusion

In this exploratory study, we found that outdoor school environments in Delhi, India needed improvement in order to potentially increase active travel and physical activity levels of children. While schools had many beneficial aspects within their environments such as trees, planted beds, and art murals, most were lacking in important safety features such as marked pedestrian crossings and traffic calming measures. Few previous studies have examined outdoor school environments and even fewer have focused on low-income countries. This study is among the first to describe school environments as they relate to children’s physical activity in a low-income country setting, and thus provides the foundation for future work in this increasingly important area of global public health [15]. Specifically, future studies could explore the relationship between enhancements and improvements to the outdoor school environment that proved lacking in this study, such as cycle lanes, pavement, school signage, and athletic tracks, and children’s physical activity. Although many of these items have been studied in high-income countries, additional data are needed on schools and children in low-middle income countries like India. Given that this is a relatively understudied area, results from this preliminary research can help inform future studies examining school environments and physical activity in children.

## Additional file

Additional file 1: SPEEDY school audit category and items with corresponding scoring. (DOCX 20 kb)

## Abbreviations

SPEEDY: The Sport, Physical Activity and Eating behavior: Environmental Determinants in Young people
DOE: Directorate of Education
