# Supplementary material for: An evaluation of outdoor school environments to promote physical activity in Delhi, India
Source: BMC Public Health. 2017 Jan 5;17:11. doi: 10.1186/s12889-016-3987-8 (PMC5217605; doi:10.1186/s12889-016-3987-8)
Supplement: Additional file 1: — SPEEDY school audit category and items with corresponding scoring. (DOCX 20 kb) [file 12889_2016_3987_MOESM1_ESM.docx]

**Additional File 1. SPEEDY school audit category and items with corresponding scoring**

| **Category and Items** | **Scoring** |
| --- | --- |
| **‘Access to the school’** | No scoring, purpose to orient data collectors to the school and school grounds |
| Locate entrance and identify if entrance for bikers, pedestrian, bus or car |  |
| Record speed limit at each entrance |  |
| **‘Surrounding area’** | Dichotomous ‘0’ (item not present) or ‘1’ (item present) |
| Space for parents to stop and drop off or pick up children |  |
| Somewhere where parents can park their cars |  |
| School bus stop |  |
| Cycle lanes:  Separated from road  On the road |  |
| Pavement:  On both sides of the road  On one side of the road only |  |
| Marked pedestrian crossing |  |
| Traffic calming |  |
| Signage:  School warning signs for road users  Road safety signs  Route signs for cyclists |  |
| **‘School grounds’** | Actual number of each item was recorded; Mean and standard deviation reported for each item; Quality of each item recorded: good, average, poor; Median value of quality reported |
| Chalk powder markings on play surfaces |  |
| Playground equipment |  |
| Pitches (marked areas with or without an incline with an associated goal or flag) |  |
| Athletic tracks |  |
| Courts |  |
| Benches |  |
| Picnic tables |  |
| Water coolers |  |
| Wildlife gardens |  |
| Uncovered cycle parking |  |
| Covered cycle parking |  |
| Assault courses (sequence of pieces of equipment designed to be used together) |  |
| Quadrangles (enclosed or semi-enclosed courtyard) |  |
| Other |  |
| Other |  |
| Other |  |
| **‘Aesthetics’** | “None” “Some” or “A lot” response options; Scored as ‘0’ ‘1’ or ‘2’ respectively but reverse coding was applied to noise, litter and graffiti items |
| Planted beds |  |
| Trees to sit under |  |
| Ambient noise |  |
| Litter |  |
| Murals or outdoor art |  |
| Graffiti |  |
| **‘Usage’** | “Very” “Somewhat” “Not at all” response options; Scored as ‘0’ ‘1’ or ‘2’ respectively |
| Sport |  |
| Informal games |  |
| General play |  |
| **‘Overall environment’** | “Strongly Agree” “Agree” “Neither” “Disagree” or “Strongly Disagree” response options; Scored -2, -1, 0, +1, +2 respectively |
| Grounds shielded from surrounding area |  |
| Grounds generally free of vandalism |  |
| Grounds generally free of vandalism |  |
| **Additional Notes** |  |
